# Supplementary material for: Monitoring cyanobacterial toxins in a large reservoir: relationships with water quality parameters
Source: PeerJ. 2019 Jul 16;7:e7305. doi: 10.7717/peerj.7305 (PMC6640620; doi:10.7717/peerj.7305)
Supplement: Supplemental Information 1 — Instrumental parameters used for ion chromatography, mass spectrometer parameters for HESI, and analyte-specific mass spectrometer settings used for cyanobacterial toxin analysis in water samples. [file peerj-07-7305-s001.docx]

**Supporting Information**

Monitoring cyanobacterial toxins in a large reservoir: Relationships with water quality parameters

Seenivasan Subbiah^†^, Adcharee Karnjanapiboonwong^†^, Jonathan D. Maul^†^, Degeng Wang^†^, Todd A. Anderson*^†^

^†^Department of Environmental Toxicology, Texas Tech University, Box 41163, Lubbock, TX 79409, USA

*Corresponding Author:

Todd A. Anderson

Department of Environmental Toxicology

Texas Tech University

Box 41163, Lubbock, TX 79409, USA

Email: [todd.anderson@ttu.edu](mailto:todd.anderson@ttu.edu)

Phone: 806-834-1587

**Table S1. Mass spectrometer parameters for HESI.**

| **Analyte** | **Precursor ion (Q1)** | **Product ion (Q3)** | **Collision Energy, (V)** | **Tube lens, (V)** |
| --- | --- | --- | --- | --- |
| Anatoxin-A | 166.1 | 131.20 | 16 | 80 |
|  |  | 149.16 | 15 | 80 |
| Microcystin-LR | 995.60 | 135.20 | 70 | 189 |
|  |  | 213.20 | 60 | 189 |
| Microcystin-LA | 910.50 | 135.00 | 52 | 172 |
|  |  | 776.20 | 12 | 172 |
| Microcystin-RR | 520.20 | 135.12 | 35 | 130 |
|  |  | 213.20 | 40 | 130 |
| Simeton | 198.12 | 124.30 | 25 | 80 |

**Table S2. Analyte specific mass spectrometer settings used for algal toxin analysis.**

| **MS operating parameters** | |
| --- | --- |
| Scan | MS/MS, SRM |
| Probe | Heated Electro Spray Ionization, HESI |
| Polarity | Positive |
| Ion Spray Voltage (v) | 3500 |
| Collision gas (Torr) | Argon, 1.5 |
| Sheath gas (Arb) | Nitrogen, 40 |
| Auxiliary gas (Arb) | Nitrogen, 10 |
| Vaporizer temperature (˚C) | 290 |
| Capillary temperature (˚C) | 350 |

**Table S3. Instrumental parameters used for ion chromatography**

| **Parameter** | **Anions** | **Cations** |
| --- | --- | --- |
| Instrument | Dionex, IC25 Ion Chromatograph with Chromeleon software | Dionex, IC25 Ion Chromatograph with Chromeleon software |
| Guard Column | Thermo Scientific, Dionex IonPac^TM^ AG14A; RFIC^TM^; 4 x 50 mm | Thermo Scientific, Dionex IonPac^TM^ CG12A; RFIC^TM^; 4 x 50 mm |
| Analytical Column | Thermo Scientific, Dionex IonPac^TM^ AS14A; RFIC^TM^; 4 x 250 mm | Thermo Scientific, Dionex IonPac^TM^ CS12A; RFIC^TM^; 4 x 250 mm |
| Column temperature | 35˚C | 40˚C |
| Eluent | 20 mM sodium hydroxide | 20 mM methanesulfonic acid |
| Flow rate | 1 mL/min | 0.8 mL/min. |
| Suppressor, SRS current | 50 mA | 50 mA |
| Total Run Time | 15 min | 20 min |
